# Country Report on Assessment of Quality of Care and Protection of Human Rights in Georgian Mental Health Institutions

**DOI:** 10.1192/j.eurpsy.2023.1153

**Published:** 2023-07-19

**Authors:** N. Zavradashvili, N. Makhashvili

**Affiliations:** 1 School of Health Sciences, The University of Georgia; 2 Arts and Sciences, Ilia State University, Tbilisi, Georgia

## Abstract

**Introduction:**

It is well established that the quality of mental health care and human rights mutually reinforcing. Until now, Georgian psychiatry is highly institutionalized, oriented towards medical treatment and suffers from a lack of recognition of the importance of the human rights concept.

**Objectives:**

The purpose of the evaluation was to gather information on the current state of human rights and service quality in the inpatient mental health facilities throughout Georgia; pilot the WHO Quality rights toolkit as a major instrument to monitor mental health institutions within the country; develop recommendations for improvement of service care in psychiatric institutions and initiate changes based on the assessment results.

**Methods:**

All inpatient mental health facilities operating in the country were selected for the evaluation. The assessment team conducted visits in facilities in March – May, 2019. All visits were planned in advance. All five themes of WHO Quality rights tool were covered. Interviews, observation and documentation reviews were used during the assessment process.

**Results:**

Infrastructure malfunction is linked to the lack of encouraging environment, with scarce of daily and social activities. Comprehensive, patient-oriented individual recovery plan has not been initiated throughout the country. Treatment is focused mainly on medication treatment aimed at reducing / removing psychotic symptoms and timely discharging patients or “calming them down“. Taking into consideration scarcity of community-based service alternatives, the patients frequently have no choice where to get the relevant service. In general, the patients are satisfied with how they are being treated. The challenge is the incidents of violence among the patients and ensuring relevant safety measures. Educational and employment programs for persons with mental disorders are not developed in the country.

**Conclusions:**

Based on the assessment findings recommendations for improvement of service care at mental health policy and institutional level were elaborated.

Despite some improvements in developing community services the assessment revealed gaps in mental health care and lack of understanding of the concept of human rights. The instrument was sensitive to identify poor treatment and violation of rights but less sensitive in determining differences in existing services. It is discussed that an in-depth assessment using the specific theme of the tool can help develop specific recommendations.

**Disclosure of Interest:**

None Declared

